# Animal Cruelty in New York City: Cruelty Cases Presented to the ASPCA in Partnership with the NYPD 2013–2022

**DOI:** 10.3390/ani15050662

**Published:** 2025-02-25

**Authors:** Shiny Caldwell, Emily Patterson-Kane, Elizabeth Brandler, Maya Gupta, Randall Lockwood

**Affiliations:** American Society for the Prevention of Cruelty to Animals, New York, NY 10018, USAelizabeth.brandler@aspca.org (E.B.); maya.gupta@aspca.org (M.G.); randall.lockwood@aspca.org (R.L.)

**Keywords:** animal cruelty, companion animals, New York City

## Abstract

Between September 2013 and 2022, the American Society for the Prevention of Cruelty to Animals (ASPCA) received 2783 suspected animal cruelty cases involving 5745 animals through a partnership with the New York City Police Department (NYPD). These cases involved dogs (2271/2783, 82%), cats (408/2783, 15%), and other species (104/2783, 4%). Dogs were most likely to be presented for suspected neglect (1424/2271, 63%), and cats for suspected non-accidental injury (233/408, 58%). Animals were most often presented by law enforcement (1018/2783, 37%), municipal shelters (383/2783, 14%), and veterinarians (311/2783, 11%). These findings contribute to understanding neglect as a commonly reported type of animal cruelty. Cruelty toward cats appears less well understood and may be under-reported or more severe, requiring further research and attention.

## Simple Summary

Between September 2013 and 2022, the American Society for the Prevention of Cruelty to Animals (ASPCA) received 2783 reports of suspected animal cruelty involving 5745 animals in partnership with the New York City Police Department (NYPD). Most cases involved dogs (2271, or 82%) and cats (408, or 15%), with dogs reported mainly for neglect (1424 cases, or 63%) and cats for suspected non-accidental injuries (233 cases, or 58%). These findings highlight the need to address neglect as a prevalent type of animal cruelty. The treatment of cats appears to be under-reported and may be more severe than currently recognized.

## 1. Introduction

Since the inception of organized municipal policing in America, starting with the creation of the Boston Police Department in 1838 and the New York Police Department (NYPD) in 1845, many resources have been dedicated to monitoring the state of society by looking in great depth at the incidence of criminal activity [1]. However, until recently, little effort has been made to formally integrate information about animal-related crimes into this picture. In addition, crimes against animals require special resources, for example, providing for the assessment, housing, care, and treatment of animal victims [2,3] and responding to associated human resource needs such as homelessness or mental health issues [4]. Objective data on animal cruelty can help communities identify the resources needed to effectively respond to these crimes and craft appropriate policies and programs.

The ASPCA-NYPD partnership program is designed to support consistent responding to suspected animal cruelty cases and is integrated with other cruelty prevention and response efforts in New York City, such as the provision of accessible veterinary services and the work of the ASPCA Community Engagement team. Data from the ASPCA-NYPD partnership provide insight into suspected cruelty cases in New York City. This information needs to be interpreted in the context of other animal cruelty data collected in the United States.

In the United States, data relating to suspected animal cruelty come piecemeal from many different sources and are often incomplete or not representative [2,5], requiring caution in analysis. Information about suspected acts of animal cruelty in the United States is available at three levels:

(1). National Data:

National averages for factors such as crime, public attitudes, and community needs are often reference points for local communities and year-on-year change. However, these data are often not representative or suitable for this purpose due to being limited in scope, incomplete, and not collected uniformly between jurisdictions.

The National Incident-Based Reporting System (NIBRS) has included animal cruelty as a reporting category since 2021 [6,7]. The FBI’s NIBRS covers 77% of the US population by jurisdiction but is limited and non-representative, with sometimes incomplete or inaccurate entries. For example, the NIBRS only includes data collected from traditional law enforcement agencies (e.g., police departments and sheriffs’ offices), and not from other agencies, such as Animal Control or SPCAs, which have the authority to enforce cruelty laws in some jurisdictions [1]. The NIBRS classifies animal cruelty as a crime against society, and therefore the information included about animal victims is minimal and difficult to access, and the species covered varies by state.

Different media monitoring organizations, such as Pet-Abuse.com and Cyberalert [8], have provided nationwide media monitoring for reports on animal cruelty cases. Media reports represent a relatively small and non-representative sample of cases, and this approach is rarely used. The most prominent site using this approach, Pet-Abuse.com https://web.archive.org/web/20060101045326/http://www.pet-abuse.com/ (accessed on 5 November 2024), ceased operations in 2006, while Cyberalert and other services only track animal cruelty-related reports when commissioned to do so by clients.

(2). Community Samples:

Data may be generated and shared by various law enforcement agencies, such as police, sheriffs, and humane law enforcement [5,9,10]. Research using these data can give insights into the characteristics of animal cruelty in a specific community but may not be generalizable. For example, Donley et al. (1999) [11] reported on all complaints received by the Massachusetts SPCA during 1996. This dataset suggests that the most common suspected cases are related to dogs and neglect; however, it is unclear whether this is true in other locations and time periods. Community samples represent a more complete sample from a large geographical area and are a way to reconcile information gained at national and case levels in order to develop generalizable knowledge about the nature of animal cruelty in the United States [12].

(3). Case Level Data:

By contrast, some datasets contain more information about a particular animal, case, or household. However, these data are typically difficult to generalize due to non-random sampling. They includes surveys and interviews, which may capture accounts of animal cruelty self-reported by perpetrators or alleged by witnesses [3], investigations of specific types of offenders, victims, or contexts [12,13], and case studies [14]. Necropsy and forensic veterinary information can be derived from pre-identified cases of suspected cruelty and broader samples that can be examined retrospectively for evidence of unreported cruelty [11]. While novel and informative, this approach to animal cruelty research is underdeveloped.

(4). Types of cruelty

The legal definitions of and penalties for animal cruelty vary by jurisdiction and depend, in part, on the severity of the act or omission. In the United States, animal cruelty is generally criminalized under state law, with penalties that can include fines, community service, probation, and incarceration. The severity of the penalties typically depends on the type of cruelty (e.g., neglect versus overt acts of cruelty), the severity of the injury caused to the animal, the perpetrator’s intent [15], and sometimes whether it involves a companion animal. Variations in the law are another factor that can limit the generalizability of community-level datasets.

New York State, for instance, criminalizes as a misdemeanor a variety of acts or omissions, intentional and not, which can include the failure to provide food, water, and necessary veterinary care to any animal, as well as acts or omissions in which unjustifiable physical pain or suffering is caused or permitted. New York State also criminalizes, as a felony offense, intentionally killing or causing serious physical injury to a companion animal where the conduct is intended to cause extreme physical pain or is carried out in an especially depraved or sadistic manner. Animal fighting-related offenses are also punishable as misdemeanors and felonies depending on the specific conduct. Failing to provide appropriate shelter to dogs outdoors is a violation (an offense for which a sentence to a term of imprisonment in excess of fifteen days cannot be imposed).

### 1.1. The Characteristics of Suspected Animal Cruelty Cases

Earlier reports of animal cruelty-related datasets suggest that in the United States the largest proportion of cases reported for suspected cruelty involve dogs [11,16,17], followed by cats and other species [11,17,18], while the most common form of cruelty, proportionately, is neglect (62–69%) [7,11,19]. Neglect includes failure to provide necessary care, whether involving single or multiple animals (including animal hoarding situations) [20].

It has been theorized that animals are more susceptible to cruelty if they are physically smaller, have a generally lower societal value, or have weaker protections, for example, animals that are young, small, free-roaming, or unowned [21,22]. On this basis, cats may generally be more vulnerable to cruelty than dogs and more likely to experience non-accidental injuries [16,23].

Reports of animal cruelty commonly involve one or a few animals [11], except for activities that often involve accumulating animals, such as hoarding and organized animal fighting. Reports on hoarding cases in the literature reflect an average of 46 animals per owner across all reports and 31 animals per owner for United States-based cases [18,24,25,26,27,28,29,30,31,32,33].

Depending on the jurisdiction, reports of suspected animal cruelty may come from various sources, such as veterinarians, animal sheltering professionals, social services workers, and concerned citizens. Traditional law enforcement agencies, as well as animal control, SPCAs, and humane societies, may be authorized to enforce cruelty laws, respond to suspected animal cruelty, and investigate these cases, which may then be prosecuted if there is sufficient evidence to support criminal charges. Veterinarians and shelter professionals encounter suspected animal cruelty in their work, as do social service agencies in the course of providing assistance to human clients. In New York State, as of 2022, veterinarians are legally mandated to report suspected cruelty to companion animals.

### 1.2. The ASPCA-NYPD Partnership

Public interest in law enforcement’s response to animal cruelty cases in New York City surged starting in 2001 with the launch of the television program Animal Precinct, a popular weekly show following the Humane Law Enforcement (HLE) Division of the ASPCA, which ran until 2008 and spawned similar programs [1]. At the peak of the show, HLE had approximately twenty sworn peace officers with authority to enforce state cruelty laws in New York City, which has a population of nearly eight million people. Animal control officers and humane law enforcement sections of private animal protection organizations with enforcement authority likewise tend to be relatively small and, in many instances, under-resourced. In addition, animal cruelty can occur alongside other serious offenses involving human victims, such as domestic violence. Crimes such as dogfighting can involve illegal drugs, illegal weapons, and other serious offenses that are outside of the scope of what animal control and humane law enforcement officers are trained to handle or have authority to investigate [34]. On the other hand, traditional law enforcement agencies that also have authority to enforce cruelty laws often lack training on investigating animal cruelty or on animal handling and care, and may not have resources for the housing, assessment, treatment, care, and potential rehoming of animals seized in connection with cruelty cases [1,35].

After a four-month pilot program in the Bronx, the ASPCA-NYPD partnership officially began in January 2014, in all five New York City boroughs [1].

As part of this partnership, the NYPD (which has approximately 36,000 officers) carries out the following:

- Responds to animal cruelty calls made to emergency (911) and nonemergency (311) police lines;

Created an animal cruelty investigation squad of specially trained officers to serve as the primary contact for animal cruelty cases;

- Investigates cases;
- Participates in community outreach efforts to encourage the public to report crimes against animals;
- Provides criminal case data to the ASPCA for analysis of animal-related crimes.

The ASPCA carries out the following:

- In coordination with the NYPD, provides training on the animal cruelty laws and investigations to all officers at different levels of command;
- Provides for the immediate care of all animals brought in by the NYPD;
- Conducts veterinary forensic exams and prepares reports on animals involved in cases where charges might be filed;
- Provides housing, care, treatment, and potential placement of animals;
- Provides legal and investigative support to NYPD officers and the five District Attorneys’ Offices in New York City;
- Participates in community events to raise awareness;
- Provides additional resources to support the NYPD’s response to animal crimes.

This report concerns suspected cases of animal cruelty where animals (alive or dead) or data relating to animals (photo, video, or testimony) were received by the ASPCA between 1 September 2013 and 31 December 2022, providing a window into incidents of suspected animal cruelty in a major metropolitan area, and an example of cases received by an animal welfare organization working in partnership with local law enforcement. The current study seeks to narrow the gap in empirical knowledge about animal cruelty through descriptive and inferential analysis of this large multi-year dataset. This dataset will build towards an understanding of animal cruelty in the United States and encourage more widespread enforcement and study, especially into poorly understood aspects of these crimes.

## 2. Materials and Methods

For this report, data were extracted from a dedicated Salesforce database that included information associated with each suspected case of animal cruelty associated with at least one live or deceased animal. Cases were identified from the date range of 1 September 2013 to 31 December 2022. Data were extracted in the form of a spreadsheet, including the variables of suspected case type, number of animals associated with the case, number and species of animals, reporting source, precinct, whether the animals were alive at intake, and whether they required humane euthanasia. Duplicate records were removed; where the same animal was associated with multiple cases, only the first case was included. Case type data were reviewed by ASPCA attorneys to ensure they were consistent with the case narrative. Other variables were reviewed by designated ASPCA staff and contractors to check for mistyped information and to recover missing values where this could be performed by reviewing the case description. Summary data were calculated within Microsoft Excel (Microsoft, 2016), and statistical testing was performed using Stata (Statacorp, StataBE 17). GIS maps were created using Esri ArcGIS Pro 3.1.2.

## 3. Results

Over the nine years in the current dataset, the ASPCA received 5745 animals, related to 2783 cases involving 5745 animals, from the NYPD. Table 1 summarizes these variables.

### 3.1. Geography

**Figure 1:**
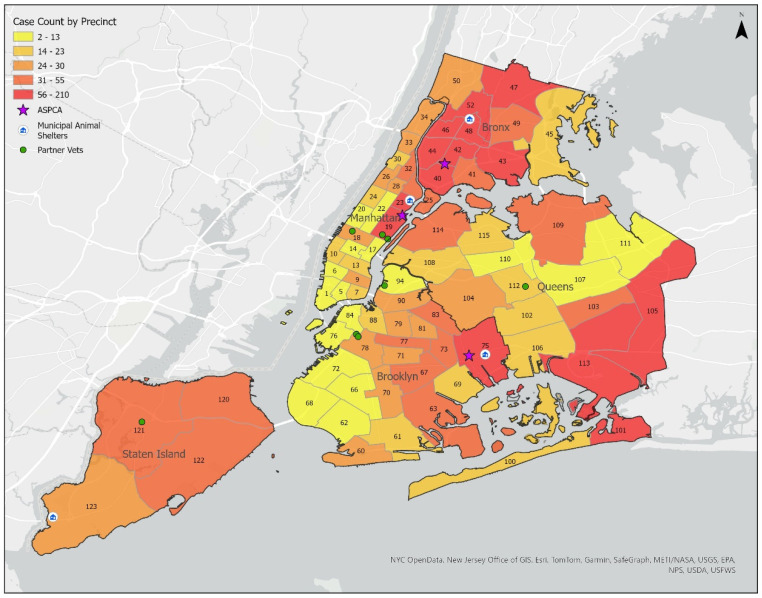
Cases of suspected animal cruelty received by the ASPCA, per precinct, between 2013 and 2022. Numbers shown on the map are precinct numbers. Figure prepared by Gregory Miller (ASPCA).

Over the 9-year study period, cases were received from all 77 precincts and 9 Housing Police Service Areas (PSAs) within the five New York City area boroughs. Figure 1 displays the geographic distribution of cases by borough and precinct. At the precinct level, more cases were received from precincts with higher populations and those proximate to ASPCA locations, municipal animal shelters, or partner hospitals (partner hospitals are those that have agreed to receive animals from law enforcement officers on behalf of the ASPCA).

### 3.2. Suspected Case Type

**Figure 2:**
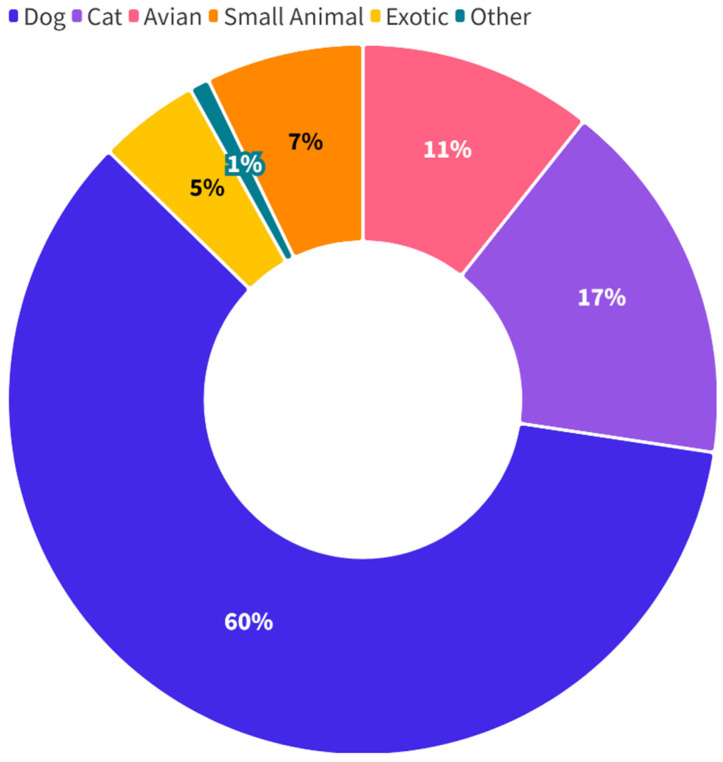
Animal species referred to the ASPCA as part of a suspected case of animal cruelty between 2013 and 2022.

Suspected cases were commonly in a category that includes neglect, hoarding (which typically involves neglect), and abandonment (1603/2783, 58%), followed by non-accidental injury (1030/2783, 43%) and organized animal fighting (33/2783, 1%). Cases with an unknown cause (117/2783, 4%) were investigations relating to animals found dead or with seriously compromised health due to unknown or unclear causes. Most suspected cases are related to dogs (2271/2783, 82%) or cats (408/2783, 15%), see Figure 2. The remaining 3% of cases were related to diverse species, including poultry and other avians, equines, other livestock, small animals, and exotic companion animals.

### 3.3. Species

**Figure 3:**
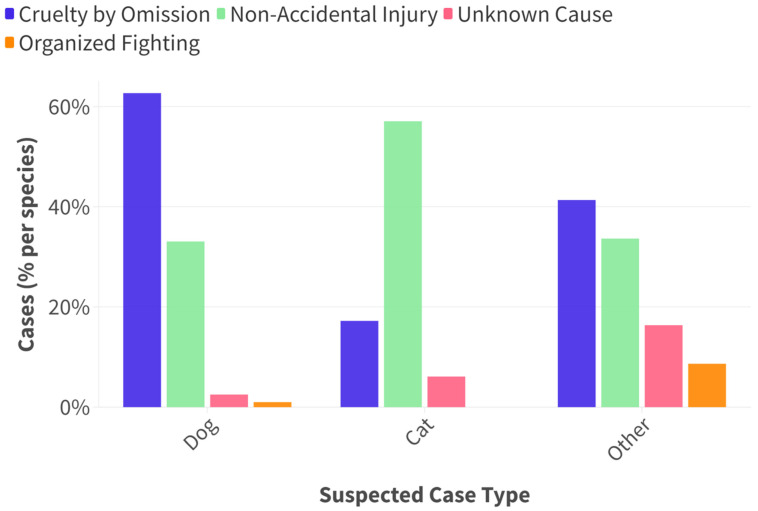
The percentage of cases presented with suspicion of specific case type. NB: fighting cases in the “other” species category related to cock fighting.

There was a relationship between species and suspected case type, with the more common suspected case type for cats being non-accidental injury, while for dogs and other species it was neglect, hoarding, or abandonment. (Figure 3.)

Binomial logistic regression was used to test whether species, case type, case size, and referral source significantly predicted the animal being dead on arrival or requiring humane euthanasia (Table 2). This analysis combined acts of omission into one category, including neglect, abandonment, and hoarding, to compare with non-accidental injury. Death as an outcome was predicted by being referred by a municipal shelter (*p* = 0.001). There was an interaction between species and suspected case type, with dogs being less likely to have death as an outcome from a suspected non-accidental injury case (*p* = 0.000017).

### 3.4. Case Size

**Figure 4:**
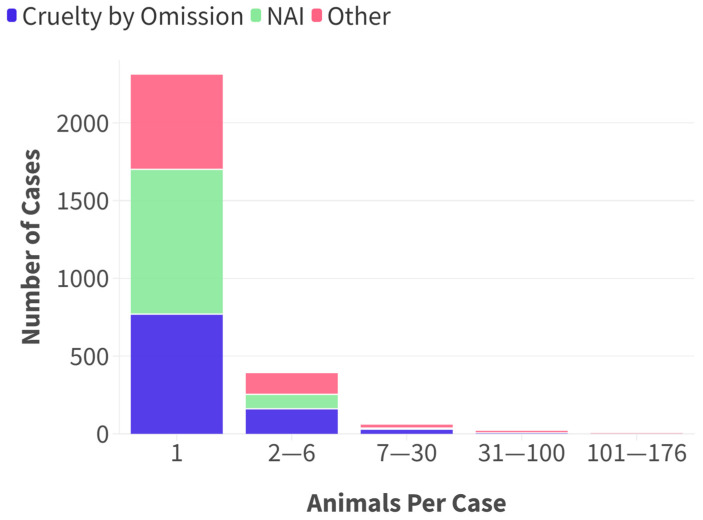
Distribution of number of animals per case within and across case types.

The median case size was one animal, with 2310/2783 (83%) cases relating to one animal (skewness = 14.23; range 1–176). (see Figure 4). Animal fighting and hoarding typically included more animals per case (Table 3 diff = −15.7432, *p* = 0.001). The suspected hoarding cases related predominantly to cats (19), dogs (4), and rabbits (1); other animals found on site included birds, fish, hamsters, and guinea pigs.

### 3.5. Referral Source

**Figure 5:**
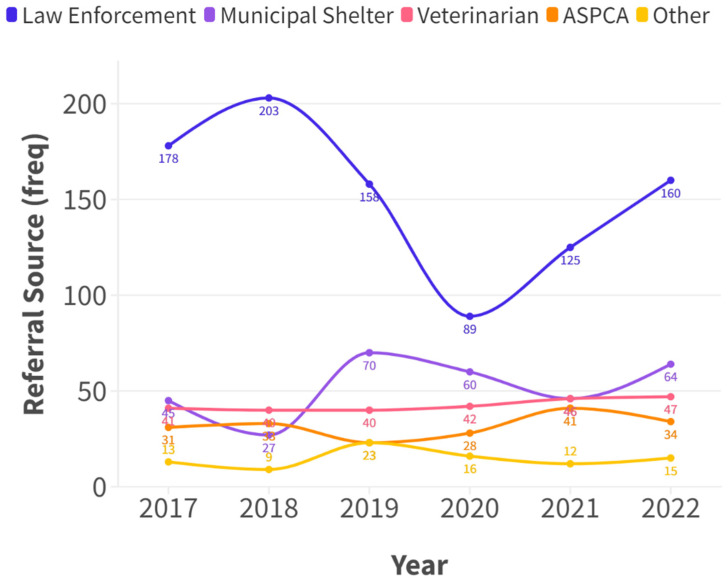
Referral source types of cases referred to the ASPCA between 2017 and 2022. “ASPCA” indicates cases originating from any ASPCA staff member, including via ASPCA veterinary facilities, mobile units, and community-based events or other activities undertaken by the ASPCA. “Other” includes cases referred from prosecutors, other sources not specified, and unknown or unidentified sources. Note, the onset of COVID restrictions in New York city occurred in 2020.

Cases were most commonly referred via law enforcement officers, including those received from the public via 311/911 or direct report to an officer (1018/2783, 37%), followed by municipal shelters (383/2783, 14%), non-ASPCA veterinarians (311/2783, 11%), and ASPCA staff (220/2783 8%). Referral source data were not captured in cases initiated before 2017, contributing to an overall 759/2783 (27%) cases with an unknown referrer. The proportion of cases received from different referral sources between 2017 and 2022 is shown in Figure 5.

The majority of veterinarian referrals came from ASPCA partner hospitals (260/280, 93%). An ASPCA partner hospital is a veterinary hospital that is open outside ASPCA hours of operation and is designated to intake animals presented by the NYPD and transfer these animals to the ASPCA. The reports shown are those initiated by hospital staff in addition to animals brought to them by law enforcement.

## 4. Discussion

Under the ASPCA-NYPD partnership, NYPD officers respond to animal cruelty complaints, other crimes with animals involved, and animals in need of assistance. Data derived from this partnership provide valuable information for replicating and extending previous findings about suspected animal cruelty in the United States and establishing metrics for initiatives in New York City.

The current data are specific to New York City; however, they are consistent with the prior literature [1,11,36]. More cases in this dataset related to suspected neglect or abandonment than non-accidental injury, and the affected animals were predominantly dogs. Neglect and abandonment were shown to be the most common types of suspected cruelty, as indicated here by the number of cases and the number of animals affected. This adds to the growing body of evidence on the prevalence of non-violent forms of cruelty [18].

Most of the cats received as part of a suspected case had injuries consistent with non-accidental causes. Researchers have suggested that cats may be especially vulnerable to violent abuse due to often being smaller than dogs [16,17] and being considered lower-status pets that receive less care than dogs. For example, cats are less likely to be taken to a veterinarian [37,38].

When interpreting this dataset, it is important to consider that not all criminal activity is witnessed, reported, or substantiated. For this reason, this dataset may paint a non-representative picture of animal cruelty in the community. For example, in the United States, approximately 30% of households have cats versus 37% having dogs. However, cat owners tend to own multiple cats, meaning Americans overall own dogs and cats in similar numbers [39]. Cats are also present in communities as unowned community cats, with estimated numbers of tens of millions nationwide [40]. Overall, it seems likely that cats are under-represented at 15% of suspected cruelty cases. In one study of 3488 animal cruelty reports, the proportion of cases that were characterized as “intentional harm” was significantly higher for cats than for dogs, and cats were killed in significantly more cases in which they were victims than dogs, including cases that involved beating, throwing, suffocation, and drowning. Cruelty to cats may be under-reported not only because of lower perceived status, but also because such cruelty may be less likely to be detected if it occurs outside the home, if the animal lacks any identification, or if an injured cat hides rather than seeking contact with an owner, as is more likely in cases of injured dogs [16]. An increase in the detection or reporting of cruelty directed towards cats and other non-dog species may dramatically change our understanding of its nature by exposing the experiences of animals in cases that are not witnessed or not reported [10].

As expected, cases of organized fighting, hoarding, and other large-scale neglect involved much larger groups of animals. Cases involving large numbers of animals require expertise, facilities, and resources that can be very demanding for animal welfare organizations, law enforcement, and prosecutors. They also represent a situation where clear and effective coordination is critical for an effective response.

Across case types, most animals were presented to the ASPCA by the NYPD responding to a complaint of alleged cruelty from a member of the public, or personally witnessing an act of alleged cruelty. The ASPCA-NYPD partnership includes resourcing and training officers to respond to crimes against animals detected by officers or reported by residents. Shelter professionals’ reporting role is also important, as shelters often receive animals with injuries or other conditions that can give rise to suspicions of cruelty. The reason for the association, in this dataset, between suspected cases coming from municipal shelters and an increased incidence of animal death remains an open question. Options include that shelter-referred animals are more seriously compromised or differences in reporting protocols. The proportion of shelter intake where cruelty would be reasonably suspected is not known, and nationwide shelter professionals’ involvement in responding to and reporting animal cruelty may be underdeveloped.

Veterinarians also have high exposure to community animals and represent the third largest number of reports. However, the veterinary community may have more untapped capacity to report. Increased reporting may also develop with the rolling out of training and policies reflecting the introduction of mandated veterinary reporting in 2022. Most reports from veterinary practices in this dataset originate from ASPCA partner hospitals that receive animals directly from law enforcement officers. However, the reports shown are those initiated by veterinary staff, excluding animals brought in by law enforcement. The higher number of reports from partner veterinary hospitals is likely to be due to the partnership encouraging the development of supportive policies and training and greater familiarity with the reporting process and law enforcement personnel. However, these veterinary hospitals may have already been more likely to generate reports given their larger size, central locations, and role in after-hours and emergency care.

Limitations of the study include the fact that ASPCA personnel initially coded suspected case types based on limited information provided by law enforcement at the time of the intake. For example, a dog may be reported abandoned by law enforcement but later discovered to have been lost or a stray. This dataset is likely to have been impacted by the changes in intake and personnel availability during the peak of the COVID-19 epidemic (2020–2022). During peak COVID precautions, reduced movements by professionals and the general public and the reduced availability of veterinary and shelter staff likely reduced their ability to encounter suspected cases of animal cruelty, which may have obscured some trends in the findings.

## 5. Conclusions

Animal welfare professionals, law enforcement, prosecutors, and veterinarians have significant and interlocking roles in preventing and responding to animal cruelty and contributing to datasets that can be used to increase the efficacy of these efforts. The current data relate to the five boroughs of New York City, and were produced via the NYPD-ASPCA partnership. These findings mirror those from other broadly comparable geographic regions [7,11], including those outside of the United States [41,42], highlighting that reported animal cruelty is most often in the form of neglect and most often in relation to dogs. The persistence of these findings across locations and data sources suggests that reports of animal cruelty have many common factors across the country and that it is a serious and entrenched problem in our communities.

Animal neglect may still be under-reported since the evidence may seem less tangible and alarming than violent forms of cruelty [43,44]. However, animal neglect may result in more prolonged suffering and may involve a large number of animals in a single case. Research illustrating the quantifiable aspects of neglect and its severe impact on animal welfare is an important aspect of cruelty research, given the prevalence of this aspect of animal cruelty.

There were comparatively few cases involving cats and other species as victims. As in other studies, cat cases involved more non-accidental injuries, which were more likely to be associated with non-live outcomes at intake. Further research is needed to elucidate why and how cats are targeted relative to dogs of similar size, specifically how cruelty to other species may differ from the more common case types relating to dogs and cruelty by omission (neglect and abandonment).

Given that North American communities largely embrace pets and show a high concern for animal welfare, the continued prevalence of animal cruelty suggests that its causes are complex and pervasive. We must employ concerted, coordinated, and innovative approaches to address the ongoing issue and reduce the future occurrence of animal cruelty. Suggestions that have been made to improve this process in other communities [45] include the following:

- Reporting mechanisms should be streamlined. The easier it is for members of the public to report suspected cruelty, the more likely they are to do it. Well-trained dispatchers should be employed who gather as much as information as possible, and then they should communicate with the most appropriate individual(s) and agency.
- Data gathering should be coordinated to ensure consistency and better tracking of all the information that might be needed to best respond to the needs of the animals and people involved.
- Alternative pathways to responding to and preventing future animal cruelty, such as through community engagement programs, should be enhanced to identify necessary assistance, support or resources.

Statistics are not the only way to measure or assess the effectiveness of animal protection work or cruelty investigations, but they are needed. Ideally, improved quantitative data collection, as well as qualitative research locally, nationally, and internationally will help agencies to better respond to the animal harm spectrum and prevent abuse and promote the wellbeing of animals and people.

## Figures and Tables

**Table 1:** Demographic and summary data for suspected cases referred to the ASPCA between September 2013 and 2022, including 2783 total cases.

Variable	Categories	Number	Percent (per capita/million)
Borough	Bronx	808	29.0% (17)
	Brooklyn	736	26.4% (31)
	Manhattan	570	20.3% (19)
	Queens	472	16.9% (27)
	Staten Island	187	6.7% (6)
	Unknown	10	0.0% (0)
Suspected Case Type	Non-Accidental Injury	1030	37.0%
	Neglect	952	34.2%
	Abandonment	627	22.5%
	Organized Animal Fighting	33	1.2%
	Hoarding	24	0.8%
	Other/Unknown	117	4.2%
Species	Dog	2271	81.6%
	Cat	408	14.6%
	Avian	29	1.0%
	Rabbit	25	0.9%
	Exotic	20	0.7%
	Wildlife	11	0.4%
	Other (incl. farm animal, equine, guinea pig, hamster)	19	0.7%
Referral Source	Law Enforcement	1018	36.6%
	Not Recorded *	758	27.2%
	Municipal Shelter **	383	13.7%
	Veterinary Hospital	310	11.1%
	ASPCA	220	7.9%
	Prosecutor	29	1.0%
	Community	14	0.1%
	Unknown cause	51	1.8%

* Referral source was not recorded for cases received prior to 2017. ** Specifically, Animal Care Centers of NYC (ACC), formerly known as Animal Care & Control of NYC, a not-for-profit corporation formed to provide animal control services in New York City.

**Table 2:** Summary table for a binary logistic regression for predictors of live versus non-live outcomes; the number of observations was 2495, chi2(7) = 132.69, and the log-likelihood = −1619.0928.

Covariate	Odds Ratio	Standard Error	z	*p* > z	Confidence Interval (95%)	
1. Dog	0.88	0.14	−0.83	0.41	0.64	1.20
1. Non-Accidental Injury	1.23	0.25	1.04	0.30	0.83	1.82
Dog vs. cat x Non-Accidental Injury	0.39	0.09	−4.30	0.001	0.25	0.60
Cruelty by Omission	1.04	0.11	0.35	0.73	0.84	1.28
Case Size	0.10	0.01	−0.62	0.54	0.98	1.01
Municipal Shelter Referral	2.17	0.28	6.07	0.001	1.69	2.79
Veterinary referral	1.09	0.15	0.65	0.52	0.84	1.43
_cons	0.80	0.13	−1.42	0.16	0.58	1.09

**Table 3:** Number of animals per case associated with different suspected case types received by the ASPCA between 2013 and 2022.

	Number of Cases	Median Animals Per Case	Minimum	Maximum
Cruelty by Omission (total)	1604	1	1	102
Neglect-other	952	1	1	52
abandonment	627	1	1	10
hoarding	24	30	8	1786
Nonaccidental injury	1031	1	1	29
Animal Fighting (total)	33	13	1	125
Dog fighting	21	8	1	60
Cock fighting	12	28	9	125

## Data Availability

Data are contained within the article.
